# A single cell death is disruptive to spontaneous Ca^2+^ activity in astrocytes

**DOI:** 10.3389/fncel.2022.945737

**Published:** 2022-07-27

**Authors:** Veronica Gomez-Godinez, Huayan Li, Yixuan Kuang, Changchen Liu, Linda Shi, Michael W. Berns

**Affiliations:** ^1^Department of Bioengineering, Institute of Engineering in Medicine, University of California, San Diego, San Diego, CA, United States; ^2^Department of Biomedical Engineering, Beckman Laser Institute and Medical Clinic, University of California, Irvine, Irvine, CA, United States; ^3^Department of Biomedical Engineering, University of California, Irvine, Irvine, CA, United States; ^4^Department of Developmental and Cell Biology, School of Biological Sciences, University of California, Irvine, Irvine, CA, United States

**Keywords:** spontaneous activity, astrocyte, photolysis, Ca^2+^, laser, cell death, calcium

## Abstract

Astrocytes in the brain are rapidly recruited to sites of injury where they phagocytose damaged material and take up neurotransmitters and ions to avoid the spreading of damaging molecules. In this study we investigate the calcium (Ca^2+^) response in astrocytes to nearby cell death. To induce cell death in a nearby cell we utilized a laser nanosurgery system to photolyze a selected cell from an established astrocyte cell line (Ast1). Our results show that the lysis of a nearby cell is disruptive to surrounding cells' Ca^2+^ activity. Additionally, astrocytes exhibit a Ca^2+^ transient in response to cell death which differs from the spontaneous oscillations occurring in astrocytes prior to cell lysis. We show that the primary source of the Ca^2+^ transient is the endoplasmic reticulum.

## Introduction

Astrocytes play important roles in the brain by maintaining homeostasis and responding to injury. In response to a wound, astrocytes are rapidly recruited so they can phagocytose damaged cells and take up molecules which may be damaging to neurons (Burda et al., 2016). Previous studies have shown that astrocytes have spontaneous Ca^2+^ activity in the CNS, in cultures, and in brain slices (Fatatis and Russell, 1992; Aguado et al., 2002; Nett et al., 2002). However, it has been shown reactive astrocytes near a stab wound in the brain no longer displayed spontaneous activity at 48hrs after injury (Aguado et al., 2002). In this study we investigate the immediate effects of death of a single cell on Ca^2+^ transients in surrounding cells.

Ca^2+^ is an important molecular messenger involved in a variety of cellular functions including gene transcription, muscle contraction, cell proliferation, and apoptosis. Cells can modulate Ca^2+^ concentrations to generate different signals by affecting the localization, duration of the elevation, and amplitude of Ca^2+^ (Bootman et al., 2001). Therefore, Ca^2+^ regulation is critical for cell function. Ca^2+^ dysregulation is thought to contribute to neurodegenerative processes and is proposed to be a target for neuroprotective agents (Fordsmann et al., 2019). Ca^2+^ dysregulation has also been shown to contribute to delayed cell damage and death following a traumatic brain injury (Weber, 2012).

We previously showed that astrocytes can respond to death of a single cell by immediately increasing their cytoplasmic Ca^2+^ concentration (Wakida et al., 2020). Here we investigate the origins of the Ca^2+^ elevation at the single cell level and the effects of a cell death on spontaneous activity of astrocytes. We found that death of a single cell is disruptive to the spontaneous Ca^2+^ activity of networked (connected) astrocytes. Further, we show that the endoplasmic reticulum (ER) stores are largely involved in the cytoplasmic elevation *via* the IP3 and Ryanodine receptors. In addition, we investigated the contribution of the NMDA receptor to determine whether glutamate from the dead cell was leading to a Ca^2+^ influx from the extracellular space.

## Materials and methods

### Cells and Ca^2+^ indicator

An established astrocyte cell line, astrocyte type-1 (Ast-1) CRL-2541 was received from ATCC. Ast-1 were grown in advanced DMEM with 2% FBS, and 1% Glutamax. Cells were incubated at 37 C with 5% CO_2_. For experiments, cells were seeded onto 35 mm glass bottom imaging dishes (Cell E&G # GBD00004-200) 3–4 days prior to experiments. To monitor intracellular Ca^2+^ levels cells were labeled with Fluo4 AM Ca^2+^ indicator (Biotium). Cells were washed with Hanks Buffered Saline Solution (HBSS) prior to the addition of 2 μM Fluo4 and 2.5 mM probenecid diluted into HBSS. Cells were incubated for 35 min at room temperature, followed by a wash with HBSS. During experiments the cells were placed back into regular medium unless otherwise specified and on an incubated stage heater set at 37 C with 5% CO2. Experiments were carried out using 50 μM 2-Aminoethyl diphenylborinate (2-APB), 2 μM Thapsigargin, 10 μM Dizocilpine (MK801), 100μM Ryanodine, or 1 μM BAPTA-AM.

### Ca^2+^ free medium

Ca^2+^ free DMEM (Gibco Thermo cat #2106828**)** was supplemented with sodium pyruvate (GibcoThermo cat# 11360) and GlutaMAX (Gibco Thermo #35050). Serum was omitted from Ca^2+^ free DMEM. A 100 mM stock EGTA solution was made by dissolving EGTA into water with a pH of 11 using NaOH. After dissolution, the 100 mM EGTA solution was pH adjusted to 7.4 with concentrated HCl. One milliliter of stock EGTA was added per 100 ml of Ca^2+^ free medium.

### Optical system

A Mai Tai Spectra Physics laser emitting 76 MHz pulses of 740 nm light was coupled to a Zeiss Axiovert 200M inverted microscope. The laser was attenuated with a Glan-Thompson polarizer and shuttered with a Uniblitz shutter controller. The beam was directed by a fast-scanning mirror as previously described and focused through a 40×, 1.3 NA objective (Duquette et al., 2018). An electron multiplying (EM) CCD camera (QuantEM:512SC, Photometrics) was utilized to visualize cells. All components of the optical system were controlled through custom Robolase software (Botvinick and Berns, 2005). A single cell was lysed per field of view by drawing a laser line through the cell at an irradiance of 1.25 × 10^12^ W/cm^2^.

### Image analysis

Fluorescence intensity quantifications were made using FIJI (FIJI is Just Image J). The background of each image was subtracted prior to quantifying the fluorescence of the cell body. All traces were normalized to the mean value before a photolysis. CaSian and MatLab were utilized to find the peak properties of each Ca^2+^ transient (Moein et al., 2018). Prism GraphPad was utilized to find statistical significance. An unpaired *t*-test was performed for comparisons between two groups. A Brown Forsythe Anova and a Dunnett's T3 were performed when more than two groups were analyzed.

## Results

### Death of a single cell disrupts astrocytic Ca^2+^ activity in surrounding cells

**Figure 1:**
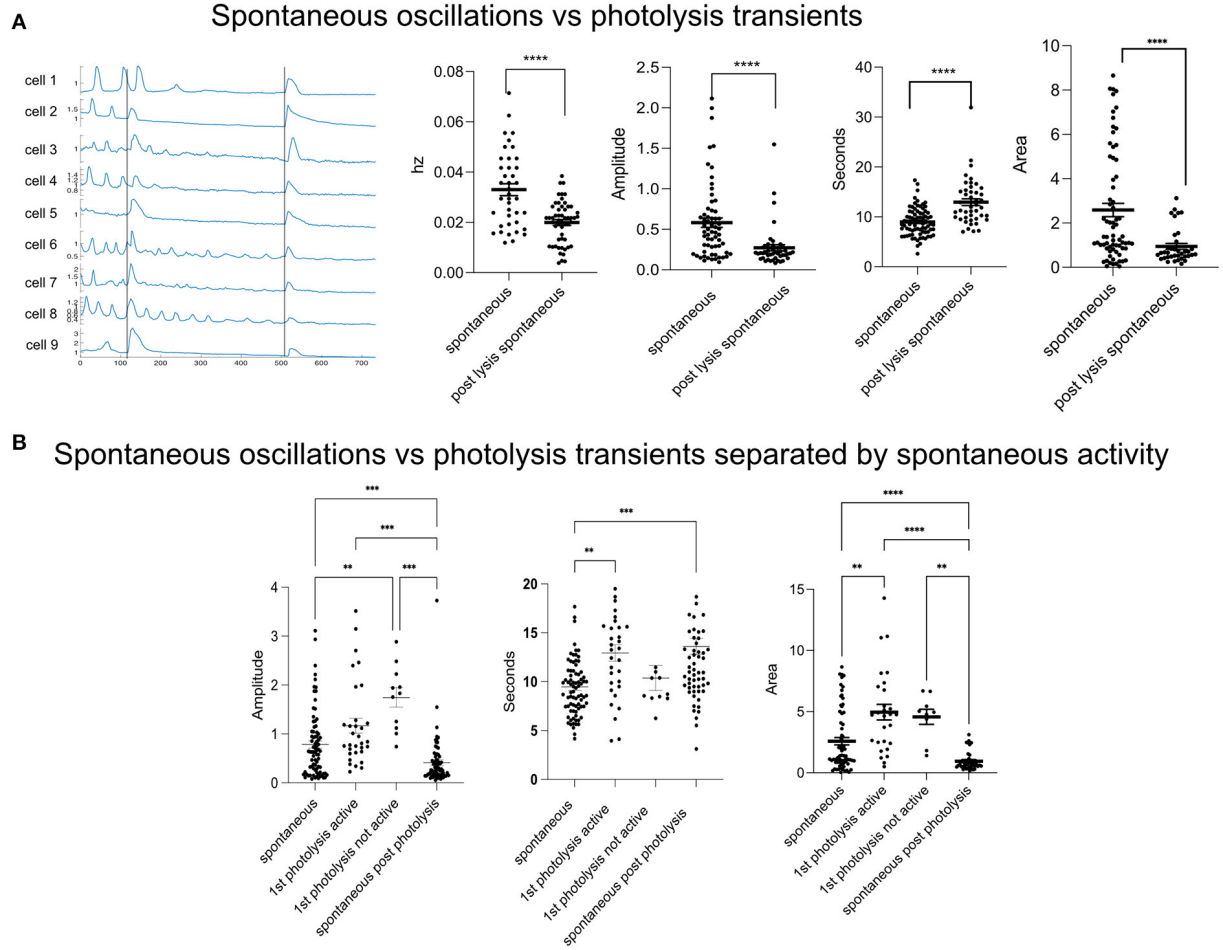
Spontaneous activity is disrupted in response to a single cell death. **(A)** Spontaneous vs. photolysis induced transients. The frequency (Hz) of spontaneous oscillations decreases following a photolysis from 0.033 + 0.02 to 0.020 + 0.001. *N* = 40 for Spontaneous and *N* = 54 for post lysis spontaneous. The amplitude and area of spontaneous oscillations also decreases after a cell death. The duration (s) increases. ****p ≤ 0.0001, ***p < 0.001, **p < 0.01. **(B)** Spontaneous oscillations vs. photolysis transients. Some cells did not show spontaneous activity before a photolysis transient and were separated and labeled as “not active.” In the graph, spontaneous refers to the Ca^2+^ characteristics of the spontaneous oscillations. First photolysis active refers to the Ca^2+^ transient in cells with previous spontaneous activity. First photolysis not active is for cells that had no previous spontaneous activity. Spontaneous post photolysis are spontaneous Ca^2+^ oscillations which occurred after a photolysis. ****p ≤ 0.0001, ***p < 0.001, **p < 0.01. Spontaneous oscillations: *N* = 81, 1st photolysis active: *N* = 33, 1st photolysis not active: *N* = 12, Spontaneous post-photolysis: *N* = 72.

The lysis of a single cell is disruptive to surrounding cells' Ca^2+^ activity. See Supplementary Video 1. Cells that had spontaneous Ca^2+^ oscillations either ceased oscillating (Figure 1A, cell 2) or had oscillations with smaller amplitudes after a nearby cell was killed (Figure 1A, cells 6 and 8). The frequency of oscillations decreased from an average of 0.033 Hz ± 0.0023–0.020 Hz ± 0.0011. Oscillations after photolysis had a smaller amplitude, and a longer duration (full width half max). Despite a longer duration, the area was smaller. Photolysis transients, cytoplasmic Ca^2+^ elevations in response to cell death, differed from spontaneous oscillations in the duration of the full width half max (FWHM) where the FWHM of the transient was 13 s + 0.62 compared to 9 s + 0.32 for spontaneous oscillations (Figure 1B). The amplitude and area under each curve were also greater for cell death induced transients.

Not all cells or fields of view showed spontaneous activity prior to photolysis. To determine whether the cell response to a cell's death was affected by spontaneous activity we separated the cells into groups of cells that had a history of spontaneous activity and those that did not. The amplitude of the photolysis transients and spontaneous activity did not differ if the cells had a history of activity prior to photolysis (Figure 1B, Amplitude). However, we found that cells with no previous Ca^2+^ activity had greater amplitudes in response to a nearby cell's photolysis when compared to cells with a history of activity prior to photolysis of a nearby cell. These results suggest that spontaneous oscillation activity is affecting the cytoplasmic Ca^2+^ increase in response to death of a nearby cell. Interestingly, transients from cells with a history of activity were longer in duration than spontaneous oscillations (Figure 1B, Seconds). Therefore, the cell does distinguish between a cell's death despite amplitudes being similar. Additionally, the area under the curve of transients was larger when compared to spontaneous activity (Figure 1B, Area).

### Inhibition of the NMDA receptor has no effect on photolysis transients

**Figure 2:**
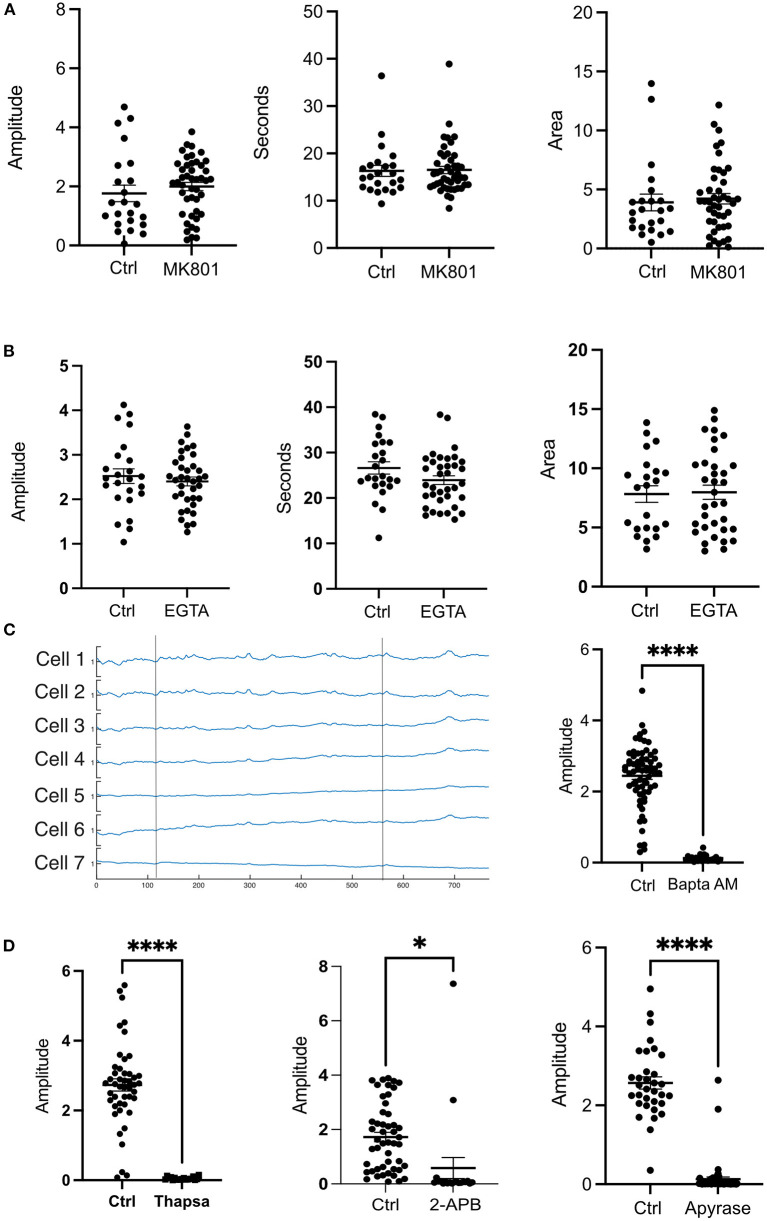
Extracellular Ca^2+^ does not contribute to the transient observed after a photolysis. **(A)** Cells were treated with the NMDAR inhibitor MK801 (10 μm). No statistical differences were found for amplitude, duration or area under the curve. Control: *N* = 23, MK801: *N* = 45. **(B)** Cells in medium without Ca^2+^ in 1M EGTA did not show any statistical differences in amplitude, duration (FWHM) or area. Control: *N* = 24, EGTA: *N* = 36. **(C)** Ca^2+^ traces of cells treated with BAPTA AM did not show a photolysis transient. Vertical lines depict the time at which a photolysis occurred. Control: *N* = 68, BAPTA AM: *N* = 23. **(D)** Treatment with Thapsigargin, 2-APB or Apyrase affected the ability of the cells to have a cytosolic Ca^2+^ transient. ****p ≤ 0.0001, *p < 0.05 Thapsigargin: Control *N* = 46, Drug *N* = 19. 2-APB: Control *N* = 49, Drug *N* = 20. Apyrase: Control *N* = 33, Drug, *N* = 56.

Various studies have shown that upon impact to the cerebral cortex, increased levels of extracellular glutamate may occur near the injury (Obrenovitch and Urenjak, 1997). Glutamate is known to interact with the NMDA receptor (NMDAR) and can lead to opening of the receptor leading to Ca^2+^ influx (Vieira et al., 2020). To determine the contribution of glutamate, and extracellular Ca^2+^ to the photolysis transient, the NMDA antagonist MK801 (Dizocilipine) was utilized. Cells in 10 μM MK801 showed no statistical differences in amplitude, duration (FWHM) and area (Figure 2A).

### The intracellular stores are largely responsible for cytosolic Ca^2+^ transients

To further investigate the contribution of extracellular Ca^2+^, cells were subjected to a photolysis in Ca^2+^ free medium with 10 mM EGTA. No significant differences were found between controls and cells in EGTA. The amplitudes, FWHM (duration), and area under the curve were similar between controls and EGTA treated cells (Figure 2B). These results indicate that intracellular Ca^2+^stores are largely responsible for the cytosolic Ca^2+^ transient in cells adjacent to the photo-lysed nearby cell. Consistent with this, treatment of cells with 10μM BAPTA Acetoxymethyl ester, an intracellular Ca^2+^ chelator, blocked Ca^2+^ fluorescent transients following photolysis. Figure 2C shows Ca^2+^ fluorescence traces of cells after photolysis. Two vertical lines depict the time at which a photolysis was induced. No transients were observed after the first or the second photolysis events.

To assess the ER contribution to the photolysis induced Ca^2+^ transient, cells were incubated with 2 μM Thapsigargin which inhibits SERCA pumps responsible for pumping Ca^2+^ back into the ER from the cytoplasm and thus leading to depletion of the ER-Ca^2+^ (Rogers et al., 1995). Treatment with Thapsigargin resulted in inhibition of a Ca^2+^ transient following photolysis (Figure 2D). Therefore, the ER is the primary source of cytosolic Ca^2+^ following photolysis of a nearby single cell.

**Figure 3:**
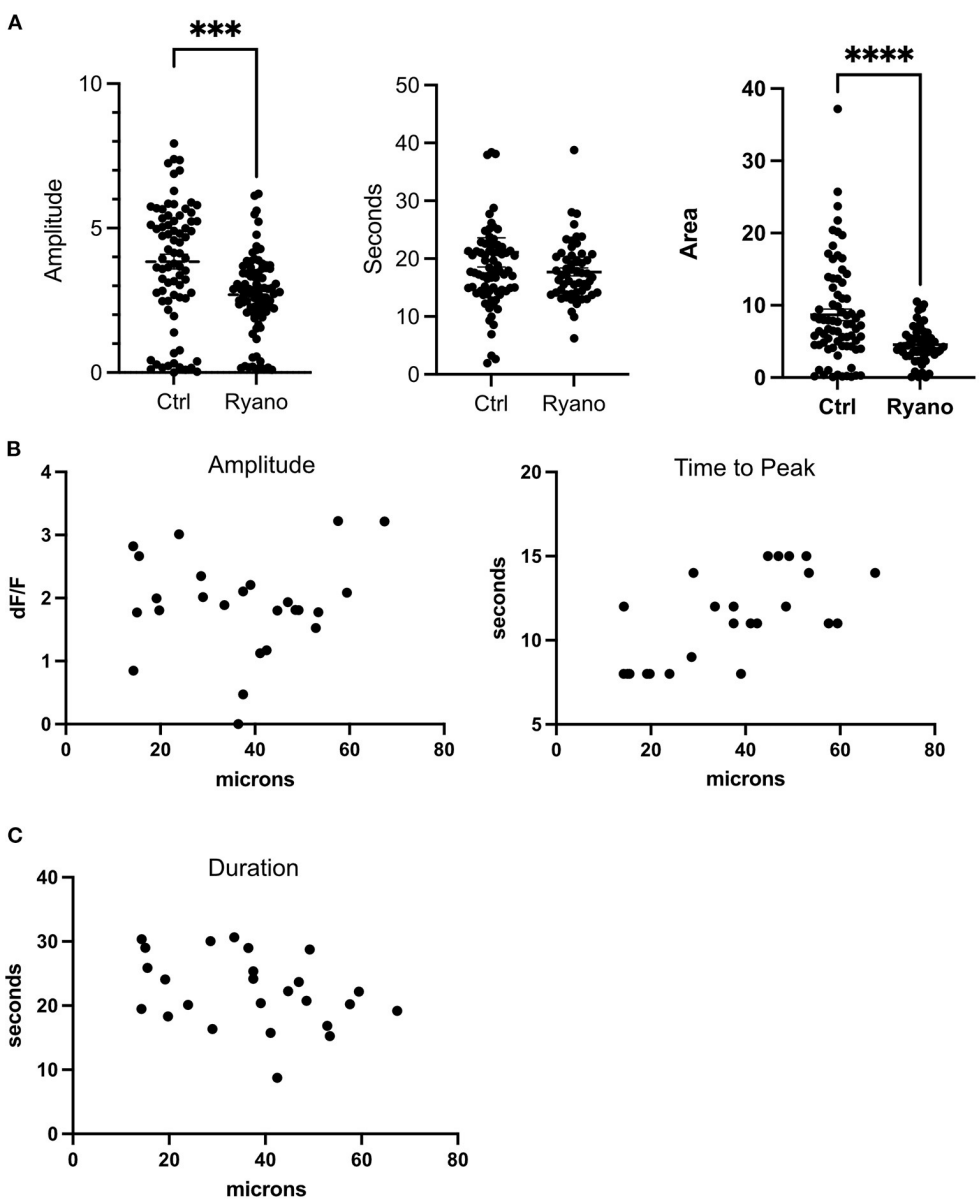
Ryanodine receptors contribute to the ER release of Ca^2+^following a photolysis. **(A)** Amplitude, duration (FWHM) and area of the transient for cells treated with Ryanodine. ****p ≤ 0.0001, ***p < 0.001 Control: *N* = 78, Ryanodine: *N* = 87. **(B)** Amplitude and time to peak plotted by distance to lysed cell. **(C)** FWHM of transient plotted by distance to lysed cell.

The inositol 1,4,5-trisphosphate (IP3) and Ryanodine receptors are responsible for Ca^2+^ release from the ER (Santulli et al., 2017). Therefore, we treated cells with 2-APB and Ryanodine to inhibit the receptors. Our results show that Ryanodine decreased the amplitude of the transient but did not abolish it like BAPTA or Thapsigargin (Figure 3A). Curiously, the duration of the transient was not affected as cells in Ryanodine had similar FWHM as cells without drugs. Nevertheless, the area under the peak was smaller for cells in Ryanodine (Figure 3A). Therefore, the Ryanodine receptors are contributing to ER Ca^2+^ release following photolysis of a nearby cell. When the cells were treated with 50 μM 2-APB the transients were almost completely abolished except for a couple of cells adjacent to the dead cell (Figure 2D). Thus, the photolysis transient is dependent on IP3.

The cells dependence on the IP3 receptors to generate the Ca^2+^ transient after a nearby cell death demonstrates that metabotropic receptors are being activated. Several studies have confirmed the presence of various metabotropic receptors in glial cells (Verkhratsky et al., 2012). ATP and glutamate can activate metabotropic receptors (Verkhratsky et al., 2012). We assessed whether ATP is responsible for ER Ca^2+^ release by treating cells with 38 units of apyrase. Apyrase catalyzes the hydrolysis of pyrophosphate bonds in ATP and ADP to form AMP (Handa and Guidotti, 1996). We found that treatment with Apyrase blocked the Ca^2+^ transient in almost all astrocytes. Similar to treatment with 50 μM 2-APB, a few cells exhibited the transient in reponse to a nearby cell death. These cells were directly adjacent to the photolyzed cell.

### The distance from the dead cell affects the time it takes a cell to reach its peak

The distances from live cells to the photolyzed cells were plotted against amplitude, time to peak, and duration (Figures 3B,C) of the Ca^2+^ transient. No correlation was identified for amplitude (df/f) and duration. However, a correlation between distance to the dead cell and the time it took a cell to reach its peak was found. Thus, cells furthest from the dead cell took longer to reach their peak. These results also indicate that the Ca^2+^ response is the same for cells quantified at distances from 14 to 67 μm from the center of the dead cell.

## Discussion

Spontaneous calcium activity in astrocytes has been reported to drive neuronal excitation by triggering NMDA receptor-mediated currents in neurons located along the wave path (Parri et al., 2001). Additionally, they have been shown to be important for neurite growth (Kanemaru et al., 2007). Therefore, the loss of spontaneous activity due to a single cell death may lead to a localized effect on neuronal excitation and a decrease in neurite growth.

A previous study showed that spontaneous activity is lost in reactive astrocytes. In that study, an area around a stab wound was monitored for Ca^2+^ activity (Aguado et al., 2002). Strikingly, we found that death of a single cell is capable of disrupting spontaneous activity in astrocytes within seconds. A decrease in Ca^2+^ transient frequency, amplitude, and change in duration were observed. The overall Ca^2+^ displacement, as measured by the area under the peak, was found to be less after photolysis of a single cell. Therefore, cell death is disruptive to Ca^2+^ activity in adjacent live cells and may signal the initiation of astrocytic reactivity.

Spontaneous activity was previously found to be dependent on both an influx from outside the cell and an efflux from the ER into the cytoplasm (Aguado et al., 2002). Therefore, it is likely that the decreases in amplitude and frequency of the spontaneous activity are due to partial depletion of the ER Ca^2+^ by transients resulting from the photolysis event. This is supported by the fact that photolysis transients are largely dependent on ER Ca^2+^ since cells treated with thapsigargin and BAPTA AM did not exhibit Ca^2+^ transients.

Ca^2+^ Transients due to a photolysis were similar in amplitude to spontaneous oscillations if the cells were previously active. Interestingly, cells differentiated the photolysis response from spontaneous activity by changing the duration of the Ca^2+^ transient; photolysis-induced transients had a longer duration than spontaneous transients. However, a larger amplitude was observed in photolysis transients in cells that lacked spontaneous activity when compared to the transients in cells with a history of spontaneous activity. These results support the hypothesis that intracellular Ca^2+^ stores are involved in the spontaneous activity and that they may be slightly depleted thus leading to a smaller amplitude when compared to cells without Ca^2+^ activity. Consistent with these observations, we found that the Ca^2+^ transient is due to an Ip3 induced ER Ca^2+^ release. However, Ryanodine receptors were also found to contribute to the photolysis transient as inhibition of the receptors lead to smaller transients. It is likely that the Ryanodine receptor is contributing through calcium induced calcium release.

We found that Ca^2+^ transients were due to ATP released from the nearby dead cell. Cells treated with Apyrase had no transients in response to nearby cell death unless the cells were directly adjacent to the lysed cell. These results are consistent with a previous study that showed ATP was responsible for the propagation of Ca^2+^ waves in astrocytes following injury to a cells membrane in a mixed culture with neurons present (Ravin et al., 2016). Our results show that a similar mechanism occurs for Ca^2+^ release following a cell death that is independent of the presence of neurons.

The role of gap junctions on ATP mediated IP3 induced Ca^2+^ release was not assessed. However, it has been demonstrated that the gap junction protein, connexin, is involved in mediating ATP release from cells to propagate calcium waves (Cotrina et al., 1998). Therefore, it is possible that the transients furthest from the dead cell are due to ATP release from undamaged cells. The correlation between time to peak and distance may be due to the propagation of calcium waves *via* this mechanism. Future studies will assess the role of gap junctions on cell death induced Ca^2+^ transients.

Within the field of view, or a distance of 70 μm from the dead cell, we did not observe any correlation with amplitude or duration and distance. However, one possibility is that cells further away and outside of the field of view do have correlations. We were unable to assess this with the objective utilized. However, the correlation between time to peak and distance is likely due to the mechanism of propagation as described in the previous paragraph.

Interestingly, the NMDA receptor was not found to participate in the Ca^2+^ transient following a cell death. Previous studies have shown increases in excitotoxic molecules, including glutamate which may activate the NMDA receptor, in response to a brain injury. However, our findings suggest that glutamate may not be extruded during a single cell death or that our cell line may not have NMDA receptors. While NMDA receptors have been documented in astrocytes (Skowronska et al., 2019), we did not assess their presence in our cell line.

In conclusion, we have shown that (1) death of a single cell is disruptive to Ca^2+^ activity in adjacent cells. (2) Spontaneous Ca^2+^ oscillations in a cell result in smaller Ca^2+^ transients following photolytic death of a nearby cell. (3) Photolysis induced Ca^2+^ transients are largely in response to the release of Ca^2+^ from the ER.

## Data availability statement

The original contributions presented in the study are included in the article/Supplementary material, further inquiries can be directed to the corresponding author/s.

## Author contributions

VG-G wrote the manuscript. VG-G, HL, YK, CL, and LS conducted experiments, analyzed data, and generated figures. MB supported the research, conceived experiments, and edited the manuscript. All authors contributed to the conception and design of the study, manuscript revision, read, and approved the submitted version.

## Funding

This material is based upon work supported by the Air Force Office of Scientific Research under award number FA9550-20-1-0052, and a gift from the Beckman Laser Institute Foundation to MB.

## Conflict of interest

The authors declare that the research was conducted in the absence of any commercial or financial relationships that could be construed as a potential conflict of interest.

## Publisher's note

All claims expressed in this article are solely those of the authors and do not necessarily represent those of their affiliated organizations, or those of the publisher, the editors and the reviewers. Any product that may be evaluated in this article, or claim that may be made by its manufacturer, is not guaranteed or endorsed by the publisher.

## Supplementary material

Supplementary Video 1 Astrocytes showing spontaneous activity in a field of view where cells are lysed at different times. Warmer colors indicate greater Ca^2+^. Lysed cells are marked with a line.

## References

[B1] Aguado F. Espinosa-Parrilla J. F. Carmona M. A. Soriano E. (2002). Neuronal activity regulates correlated network properties of spontaneous calcium transients in astrocytes in situ. J. Neurosci. 22, 9430–9444. 10.1523/JNEUROSCI.22-21-09430.2002 12417668 PMC6758057

[B2] Bootman M. D. Collins T. J. Peppiatt C. M. Prothero L. S. MacKenzie L. Smet P. D.. (2001). Calcium signalling–an overview. Semin. Cell Dev. Biol. 12, 3–10. 10.1006/scdb.2000.0211 11162741

[B3] Botvinick E. L. Berns M. W. (2005). Internet-based robotic laser scissors and tweezers microscopy. Microsc. Res. Tech. 68, 65–74. 10.1002/jemt.20216 16228982

[B4] Burda J. E. Bernstein A. M. Sofroniew M. V. (2016). Astrocyte roles in traumatic brain injury. Exp. Neurol. 275(Pt 3), 305–315. 10.1016/j.expneurol.2015.03.020 25828533 PMC4586307

[B5] Cotrina M. L. Lin J. H. Alves-Rodrigues A. Liu S. Li J. Azmi-Ghadimi H.. (1998). Connexins regulate calcium signaling by controlling ATP release. Proc Natl Acad Sci U. S. A. 95, 15735–15740. 10.1073/pnas.95.26.15735 9861039 PMC28113

[B6] Duquette M. L. Kim J. Shi L. Z. Berns M. W. (2018). LSD1 mediated changes in the local redox environment during the DNA damage response. PLoS ONE 13, e0201907. 10.1371/journal.pone.0201907 30096172 PMC6086436

[B7] Fatatis A. Russell J. T. (1992). Spontaneous changes in intracellular calcium concentration in type I astrocytes from rat cerebral cortex in primary culture. Glia 5, 95–104. 10.1002/glia.440050203 1349589

[B8] Fordsmann J. C. Murmu R. P. Cai C. Brazhe A. Thomsen K. J. Zambach S. A.. (2019). Spontaneous astrocytic Ca(2+) activity abounds in electrically suppressed ischemic penumbra of aged mice. Glia 67, 37–52. 10.1002/glia.23506 30427548

[B9] Handa M. Guidotti G. (1996). Purification and cloning of a soluble ATP-diphosphohydrolase (apyrase) from potato tubers (*Solanum tuberosum*). Biochem. Biophys. Res. Commun. 218, 916–923. 10.1006/bbrc.1996.0162 8579614

[B10] Kanemaru K. Okubo Y. Hirose K. Lino M. (2007). Regulation of neurite growth by spontaneous Ca2+ oscillations in astrocytes. J. Neurosci. 27, 8957–8966. 10.1523/JNEUROSCI.2276-07.2007 17699677 PMC6672170

[B11] Moein M. Grzyb K. Goncalves Martins T. Komoto S. Peri F. Crawford A. D.. (2018). CaSiAn: a Calcium Signaling Analyzer tool. Bioinformatics 34, 3052–3054. 10.1093/bioinformatics/bty281 29668830 PMC6129310

[B12] Nett W. J. Oloff S. H. McCarthy K. D. (2002). Hippocampal astrocytes in situ exhibit calcium oscillations that occur independent of neuronal activity. J. Neurophysiol. 87, 528–537. 10.1152/jn.00268.2001 11784768

[B13] Obrenovitch T. P. Urenjak J. (1997). Is high extracellular glutamate the key to excitotoxicity in traumatic brain injury? J. Neurotrauma 14, 677–698. 10.1089/neu.1997.14.677 9383088

[B14] Parri H. R. Gould T. M. Crunelli V. (2001). Spontaneous astrocytic Ca2+ oscillations in situ drive NMDAR-mediated neuronal excitation. Nat. Neurosci. 1, 803–812. 10.1038/90507 11477426

[B15] Ravin R. Blank P. S. Busse B. Ravin N. Vira S. Bezrukov L.. (2016). Blast shockwaves propagate Ca(2+) activity via purinergic astrocyte networks in human central nervous system cells. Sci. Rep. 6, 25713. 10.1038/srep25713 27162174 PMC4861979

[B16] Rogers T. B. Inesi G. Wade R. Lederer W. J. (1995). Use of thapsigargin to study Ca2+ homeostasis in cardiac cells. Biosci. Rep. 15, 341–349. 10.1007/BF01788366 8825036

[B17] Santulli G. Nakashima R. Yuan Q. Marks A. R. (2017). Intracellular calcium release channels: an update. J. Physiol. 595, 3041–3051. 10.1113/JP272781 28303572 PMC5430224

[B18] Skowronska K. Obara-Michlewska M. Zielinska M. Albrecht J. (2019). NMDA receptors in astrocytes: in search for roles in neurotransmission and astrocytic homeostasis. Int. J. Mol. Sci. 20, 309. 10.3390/ijms20020309 30646531 PMC6358855

[B19] Verkhratsky A. Rodriguez J. J. Parpura V. (2012). Calcium signalling in astroglia. Mol. Cell. Endocrinol. 353, 45–56. 10.1016/j.mce.2011.08.039 21945602

[B20] Vieira M. Yong X. L. H. Roche K. W. Anggono V. (2020). Regulation of NMDA glutamate receptor functions by the GluN2 subunits. J. Neurochem. 154, 121–143. 10.1111/jnc.14970 31978252 PMC7351600

[B21] Wakida N. M. Gomez-Godinez V. Li H. Nguyen J. Kim E. K. Dynes J. L.. (2020). Calcium dynamics in astrocytes during cell injury. Front. Bioeng. Biotechnol. 8, 912. 10.3389/fbioe.2020.00912 32984268 PMC7481337

[B22] Weber J. T. (2012). Altered calcium signaling following traumatic brain injury. Front. Pharmacol. 3, 60. 10.3389/fphar.2012.00060 22518104 PMC3324969

